# Quercetin prevents small intestinal damage and enhances intestinal recovery during methotrexate-induced intestinal mucositis of rats

**DOI:** 10.29219/fnr.v62.1327

**Published:** 2018-03-28

**Authors:** Igor Sukhotnik, Dalia Moati, Ron Shaoul, Boaz Loberman, Yulia Pollak, Betty Schwartz

**Affiliations:** 1 The Bruce Rappaport Faculty of Medicine, Technion-Israel Institute of Technology, Laboratory of Intestinal Adaptation and Recovery, Haifa, Israel; 2 Department of Pediatric Surgery, Bnai Zion Medical Center, Haifa, Israel; 3 Pediatric Gastroenterology and Nutrition Unit, Meyer Children’s Hospital of Haifa, Rambam Medical Center, Haifa, Israel; 4 Institute of Biochemistry, Food Science and Nutrition, The Robert H. Smith Faculty of Agriculture, Food and Environment, The Hebrew University of Jerusalem, Rehovot, Israel

**Keywords:** chemotherapy, methotrexate, mucositis, quercetin, enterocyte proliferation, enterocyte apoptosis

## Abstract

**Background:**

Gastrointestinal mucositis occurs as a consequence of cytotoxic treatment. Quercetin (QCT) is a bioflavonoid that exerts significant antioxidant activity and anti-inflammatory as well as anti-malignancy properties.

**Objective:**

To evaluate the effects of oral QCT consumption in preventing intestinal mucosal damage and stimulating intestinal recovery following methotrexate (MTX)-induced intestinal damage in a rat model.

**Design:**

Male Sprague–Dawley rats were divided into four groups: Control Group A (CONTR) – rats were treated with 2 cc of saline given by gavage for 6 days. Group B (CONTR-QCT) – rats were treated with QCT (100 mg/kg in 2 ml saline) given by gavage 3 days before and 3 days after intraperitoneal (IP) injection of saline. Group C (MTX) – rats were injected a single dose (25 mg/kg) of MTX IP. Group D (MTX-QCT) rats were treated with QCT (similar to Group B) 3 days before and 3 days after IP MTX injection. Intestinal mucosal parameters (bowel and mucosal weight, mucosal DNA and protein content, and villus height and crypt depth), enterocytes proliferation, and enterocyte apoptosis degree were investigated at sacrifice on the 4th day after MTX or saline injection.

**Results:**

Administration of QCT to MTX-treated rats resulted in: (1) significant decrease in intestinal injury score, (2) significant increase in intestinal and mucosal weight in jejunum and ileum, (3) increase on the protein content of the ileum, (4) increase in the villus height in the ileum, (5) increase of crypt depth of jejunum and ileum, and (6) increase in cell proliferation in the jejunum and ileum compared to MTX-nontreated group.

**Conclusions:**

Administration of QCT prevents intestinal damage and improves intestinal recovery following MTX-induced intestinal damage in a rat. We surmise that the effect of QCT is based on induction of cell proliferation in the crypt rather than inhibition of apoptosis.

Chemotherapy-induced mucositis occurs in 10–40% of patients undergoing chemotherapy for cancer treatment, leads to dose reduction or prevention of continuation of selected therapies, prolongs hospital stay, increases re-admission rates, increases healthcare cost, compromises patients’ nutritional status, impairs patients’ quality of life, and is occasionally fatal (1–3). Mucositis is clinically manifested by oral symptoms such as pain and erythema, and gastrointestinal symptoms such as bloating, abdominal pain, and diarrhea. Despite advances in the understanding of mucositis over recent years, the underlying mechanisms of the condition and it impact on intestinal structure and function are poorly understood. Since the intestinal epithelium is a highly proliferative tissue, chemotherapy may result in direct intestinal cellular injury similar to the tumor cell targeting strand breaks of the DNA (4). In addition, chemotherapy may exert a cell-damaging or cell-destroying effect through the generation of reactive oxygen species (5) or through enzymatic pathways or activation of transcription factors (NF-κB), which leads to upregulation of genes responsible for the production of pro-inflammatory cytokines such as TNF-α, IL-1β, and IL-6. Cumulatively, these activities lead to tissue injury and apoptosis (6).

Methotrexate (MTX) is the most widely used antimetabolite in clinical oncology and is effective in the treatment of acute lymphocytic leukemia (ALL), non-Hodgkin’s lymphoma, histiocytosis, and osteosarcoma (5). The primary action of MTX is inhibition of DNA synthesis due to its nature of being a folic acid analogue, by binding to the enzyme dihydrofolate reductase. This leads to the inhibition of proliferation of cells populating the crypts of the small intestine (7).

Quercetin (QCT) (3,3',4',5,7-pentahydroxyflavone) is a highly ubiquitous and well-classified dietary flavanol found in various fruits, vegetables, and other plant products including onions, broccoli, kale, oranges, blueberries, apples, and tea. QCT was demonstrated to exert beneficial effects on many disorders including diabetes, depression, and hypertensive conditions (8). QCT has demonstrated antiproliferative and proapoptotic activity in various cancer cell types. QCT is readily metabolized by tyrosinase into various compounds that promote anticancer activity (9). It prevents oxidative injury and cell death by various mechanisms including oxygen radical scavenging activity, inhibiting xanthene oxidase, lipid peroxidation, and chelating metal ions (10). It has also been reported that QCT inhibits the antigen-immunoglobulin E mediated tumor necrosis factors-α and IL-4 production in Type I allergic reactions and concomitantly decreases the expression of Th2-type cytokines (IL-4, IL-13, and IL-5) by basophils (11).

The purpose of this study was to evaluate the effects of oral QCT consumption in preventing intestinal mucosal damage caused by MTX in a rat model and to determine the mechanisms by which QCT influences enterocyte turnover following MTX-induced damage, including its effect on enterocyte proliferation and death via apoptosis.

## Materials and methods

### Animals and experimental design

The experiments described and animal care were all conducted in compliance with the guidelines established by the *Guide for the Care and Use of Laboratory Animals*, Rappaport Faculty of Medicine, Technion (Haifa, Israel). Male Sprague-Dawley rats (250–300 g) were used in this study. The rats were housed under standardized conditions (12 h light-dark cycle, controlled room temperature) for 5–7 days.

### Experimental design

Rats were divided randomly into four experimental groups. Control (CONTR) rats (Group A) were treated with oral normal saline (2 ml) given by gavage for 6 days with intraperitoneal (IP) saline injection at day 3; CONTR-QCT rats (Group B) were treated with QCT (Sigma-Aldrich, Rehovot, Israel) given by gavage at a dose of 100 mg/kg/2 ml saline for 6 days and saline IP injection at day 3; MTX rats (Group C) were treated with one dose (25 mg/kg) of MTX (Sigma-Aldrich, Rehovot, Israel) administered IP; and MTX-QCT rats (Group D) were treated with oral QCT (similar to Group B) given by gavage 72 h before and 72 h after IP injection of MTX. The dose of MTX and QCT was chosen in accordance with the literature data. In a recent study, Sangild et al. summarized data about animal models of chemotherapy-induced mucositis with respect to anticancer drugs, dose, kinds of animals, clinical manifestation, and pathophysiology (12). In a recent study, Singh et al. investigated the effects of QCT in preventing nonsteroidal anti-inflammatory drugs (NSAID)-induced small intestinal damage.The effects of different doses (35, 50, and 100 mg kg^−1^ PO) were compared and the best positive effect was observed at the doses of 50 and 100 mg kg^−1^ of oral QCT (13).

Seventy-two hours after MTX or saline injection, the animals were anesthetized with pentobarbital (75 mg/kg, IP) and were sacrificed. A sample of 10 cm of proximal jejunum and 10 cm of distal ileum was taken for investigation. The intestine was split on the antimesenteric border, washed with cold saline, and dried, and each segment was weighed. The mucosa was scrapped from the underlying tissue with a glass slide. Mucosal samples were homogenized with TRIzol reagent (Sigma-Aldrich, Rehovot, Israel). DNA and protein were extracted by the method of Chromsczynski (14). An amount of 100 mg tissue was mixed with 1 ml of TRIzol reagent and homogenized for 2 min. After spinning, the supernatant was mixed with 0.2 ml chloroform. After 3 min of spinning the content was separated into three phases. DNA was isolated from the interphase using ethanol, washed with sodium citrate, and stabilized with 75% ethanol. Protein was isolated from the lower phase using isopropyl alcohol, washed with guanidine hydrochloride, and stabilized with 100% ethanol. Quantitation of DNA was performed by spectrophotometry using the A260 value (one A260 unit equals 50 μg of double-stranded DNA). Concentration of the final protein concentration was detected using the Bio-Rad protein assay technique. DNA and protein levels were expressed as micrograms per centimeter of bowel per 100 g of body weight.

### Intestinal histology

Intestinal samples from the proximal jejunum and distal ileum were fixed in buffered 4% formalin, dehydrated in progressive concentrations of ethanol, cleared in xylene, and embedded in paraffin wax. Deparaffinized 5 μm sections were stained with hematoxylin and eosin. The mucosal damage of the small bowel was graded using an intestinal injury score as described by Kesik et al. (15). The following parameters were investigated in the jejunum and ileum: (1) degeneration of surface and crypt epithelium, (2) degeneration of villus structure and vacuolization in the surface epithelium, and (3) inflammatory cell infiltration and edema in the lamina propria. For each parameter, a score was given using a semiquantitative scale as follows: 0 = none, 1 = mild, 2 = moderate, and 3 = severe, giving a maximum possible score of 9 for each intestinal region.

Ten villi and crypts were selected for the villus height and crypt depth measurements. Histological images were loaded on a 760 × 570 pixels resolution buffer using a computerized image analysis system composed of a trichip RGB video-camera (Sony, Japan), installed on a light microscope (Zeiss, Germany), and attached to an IBM compatible personal computer (Pentium III, MMX, 450 MHz, 125 MB RAM), equipped with a frame grabber. Images were captured, digitized, and displayed on a high-resolution 17-inch color monitor. The villus height and crypt depth were measured using the Image Pro Plus 4 image analysis software (Media Cybernetics, Baltimore, MD,).

### Enterocyte proliferation and apoptosis

Crypt cell proliferation was assessed using 5-bromodeoxyuridine (5-BrdU). Standard BrdU labeling reagent (Zymed Laboratories, Inc, San Francisco, CA) was injected intraperitoneally at a concentration of 1 ml/100 g body weight 2 h before sacrifice. Tissue slices (5 μm) were stained with a biotinylated monoclonal anti-BrdU antibody system provided in a kit form (Zymed Laboratories, Inc, San Francisco, CA). An index of proliferation was determined as the ratio of crypt cells staining positively for BrdU per 10 crypts.

Additional 5 μm thick sections were prepared to establish the degree of enterocyte apoptosis. Immunohistochemistry for Active Caspase-3 (Caspase-3 cleaved concentrated polyclonal antibody; dilution 1:100; Biocare Medical, Walnut Greek, CA) was performed for identification of apoptotic cells using a combination of the streptavidin-biotin-peroxidase method and microwave antigen retrieval on formalin-fixed, paraffin-embedded tissues according to the manufacturer’s protocols. The apoptotic index (AI) was defined as the number of apoptotic cells per 1,000 cells/10 villi.

A qualified pathologist blinded as to the source of intestinal tissue performed all measurements.

### Western blotting

The tissues were homogenized in RIPA lysis buffer containing 50 mM Tris–HCl (pH 7.4), 150 mM NaCl, 1% NP-40, and 2 mM ethylenediaminetetraacetic acid (EDTA), supplemented with a cocktail of protease and phosphatase inhibitors. Protein concentrations were determined by Bradford reagent according to the manufacturer’s instructions. Samples containing equal amounts of total protein (30 μg) were resolved by SDS-PAGE under reducing conditions. After electrophoresis, proteins were transferred to a polyvinylidene difluoride (PVDF) membrane (Bio-Rad Laboratories) and probed with primary antibodies to anti-phospho-ERK antibody (1:2,500 dilution, sc-7383), anti-caspase-3 antibody (1:1,000 dilution, AM20-100 ug Oncogen), and monoclonal anti-γ tubulin antibody (1:5,000 dilution, Sigma T6557). Horseradish peroxidase-conjugated secondary antibody from Jackson ImmunoResearch Laboratories Inc. (West Grove, PA) and an enhanced chemiluminescent substrate from Bio-Rad Laboratories (Hercules, CA,) were purchased.

### Statistical analysis

The data are expressed as the mean ± SEM. Statistical analysis of intestinal mucosal parameters, enterocyte proliferation, and apoptosis was performed using the one-way analysis of variance (ANOVA) test, followed by the unpaired student’s *t*-test, with *p* less than 0.05 considered statistically significant. Prism software was used (GraphPad Software, Inc., San Diego, CA) and statistical significance was defined as *p* < 0.05.

## Results

### Body weight changes

**Fig. 1:**
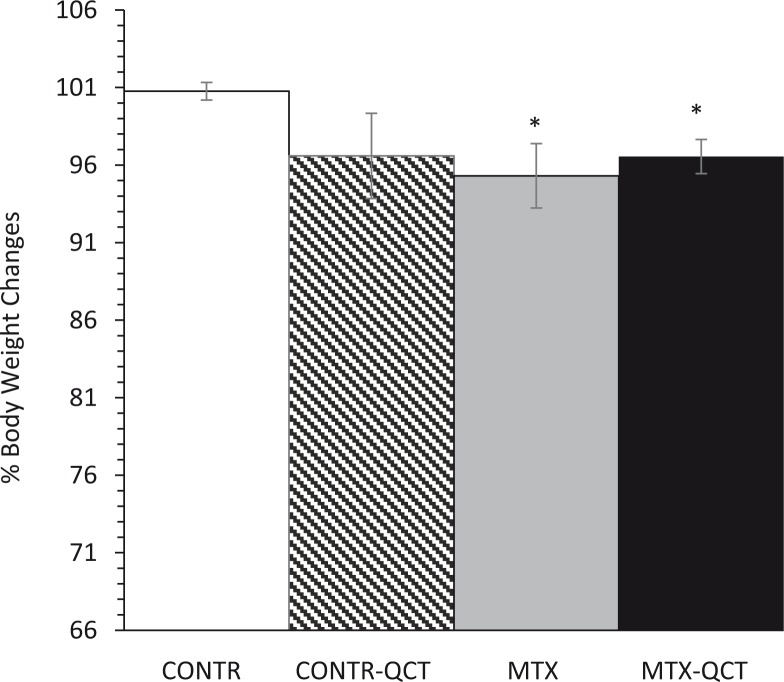
Effects of MTX and quercetin on body weight changes. Values are mean ± SEM. CONTR, control; MTX, methotrexate; QCT, quercetin. *p < 0.05 MTX and MTX-QCT versus CONTR rats.

Although CONTR-QCT (Group B) rats demonstrated a trend toward a decrease in body weight compared to CONTR animals (Group A), the trend was not statistically significant. MTX rats (Group C) demonstrated a significant decrease in final body weight (95 ± 2 vs. 101 ± 0.6% initial body weight, *p* < 0.05) compared to CONTR animals (Group A) (Fig. 1). Oral QCT supplementation in MTX-treated rats (Group D) resulted in a trend toward an increase in final body weight compared to MTX animals (Group C); however, this trend was not statistically significant.

### Intestinal mucosal parameters

Treatment of CONTR rats with QCT (Group B) did not change significantly intestinal mucosal parameters compared to CONTR animals (Group A) (Table 1). MTX rats (Group C) showed a significant decrease in bowel weight in jejunum (*p* < 0.05) and ileum (*p* < 0.05), mucosal weight in jejunum (*p* < 0.05) and ileum (*p* < 0.05), mucosal DNA in jejunum (*p* < 0.05) and ileum (*p* < 0.05), and mucosal protein in jejunum (*p* < 0.05) and ileum (*p* < 0.05) compared to CONTR animals (Group A). Administration of QCT (Group D) resulted in a significant increase in ileal (*p* < 0.05) bowel weight, jejunal (*p* < 0.05) mucosal weight, jejunal (*p* < 0.05) and ileal (*p* < 0.05) mucosal DNA, and jejunal (*p* < 0.05) and ileal (*p* < 0.05) mucosal protein compared to MTX non-treated animals (Group C).

**Table 1:** Intestinal mucosal parameters

	CONTR	CONTR-QCT	MTX	MTX-QCT
Bowel weight (mg/cm/100 g of body weight)				
Jejunum	20.6±0.6	18.8±1.2	15.7±0.5^*^	16.7±0.4^*^^#^
Ileum	19.8±0.5	18.9±1.0	16±0.6^*^	17.6±0.6^*^^#^
Mucosal weight (mg/cm/100 g of body weight)				
Jejunum	9.4±0.4	8.9±0.5	6.6±0.7^*^	7.7±0.3^*^^#^
Ileum	9.3±0.2	7.4±0.2	6.7±0.9^*^	7.6±0.4^*^^#^
Mucosal DNA (μg/cm/100 g of body weight)				
Jejunum	37±5	25±3	15±2^*^	24±3^*^^#^
Ileum	30±3	20±2	13±1^*^	22±3^*^^#^
Mucosal protein (μg/cm/100 g of body weight)
Jejunum	34±3	38±6	27±5^*^	41± 5^*^^#^
Ileum	34±1	33±1	24±3^*^	40±4^*^^#^

CONTR, control; QCT, quercetin; MTX, methotrexate. Values are Mean ± SEM.

* *p* < 0.05 all groups versus control group

# *p* < 0.05 MTX-QCT versus MTX.

### Microscopic bowel appearance

**Fig. 2:**
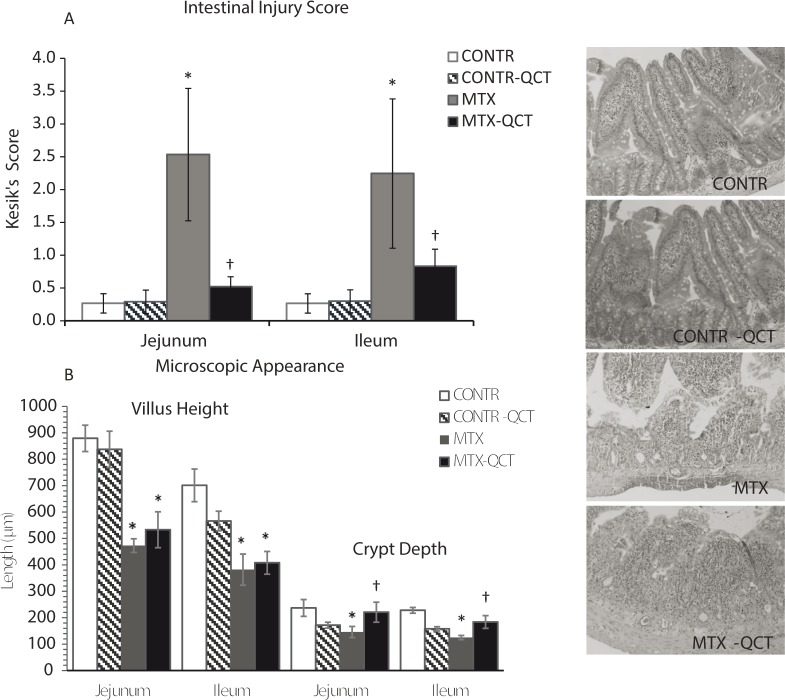
Effects of MTX and QCT on intestinal injury score and microscopic appearance. The following parameters were investigated: (A) Degeneration of surface and crypt epithelium; (B) degeneration of villus structure, vacuolization in the surface epithelium; and (C) inflammatory cell infiltration, and bleeding and edema in the lamina propria. For each criterion, a score was given using a semiquantitative scale as follows: 0 = none, 1 = mild, 2 =moderate, 3 = severe, giving a maximum possible score of 9 for each segment. Values are mean ± SEM. CONTR, control; MTX, methotrexate; QCT, quercetin. *p < 0.05 MTX and MTX-QCT versus CONTR rats; ^†^*p* < 0.05 MTX-QCT versus MTX.

Treatment of CONTR rats with QCT (Group B) did not change significantly intestinal injury score compared to CONTR animals (Fig. 2A). MTX-induced intestinal damage (Group C) resulted in a significant increase in Keisik’s intestinal injury score in jejunum (*p* < 0.05) and ileum (*p* < 0.05) compared to CONTR animals (Group A). Treatment of MTX rats with QCT (Group D) resulted in a significant decrease in intestinal injury score in jejunum (*p* < 0.05) and ileum (*p* < 0.05) compared to MTX animals (Group C).

MTX rats (Group C) exhibited a significant decrease in villus height in jejunum (*p* < 0.05) and ileum (*p* < 0.05) as well as in crypt depth in jejunum (39%, *p* < 0.05) and ileum (*p* < 0.05) compared to CONTR animals (Group A) (Fig. 2B). MTX-QCT animals (Group D) demonstrated a significant increase in jejunal (*p* < 0.05) and ileal (*p* < 0.05) crypt depth compared to MTX animals (Group D) as well as a trend toward an increase in villus height in jejunum and ileum; however, this trend was not statistically significant.

### Enterocytes proliferation

**Fig. 3:**
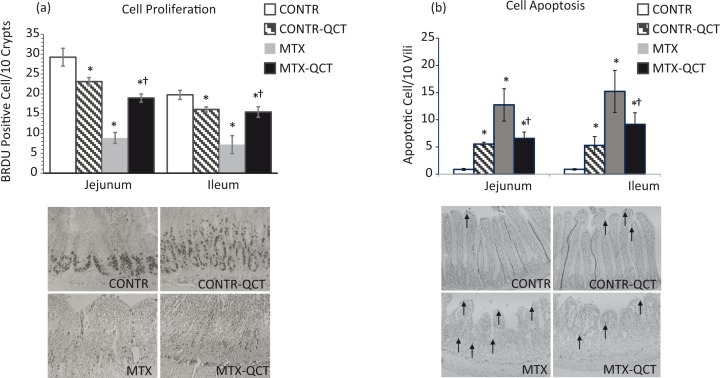
Effects of MTX and oral QCT on cell proliferation and apoptosis. The number of BrdU-labeled cells in 10 well-oriented, longitudinal crypts per section from each rat was determined using light microscopy. Identification of apoptotic cells was performed using immunohistochemistry for Caspase-3. The apoptotic index is expressed as the percentage of apoptotic cells per 10 villi. Values are mean ± SEM. CONTR, control; MTX, methotrexate; QCT, quercetin. *p < 0.05 MTX and MTX-QCT versus CONTR rats; ^†^*p* < 0.05 MTX-QCT versus MTX.

CONTR-QCT (Group B) demonstrated a small but significant decrease in the rate of cell proliferation compared to CONTR experimental group (Group A) (Fig. 3). MTX-induced intestinal damage resulted in a significant inhibition of cell proliferation in jejunum (*p* < 0.001) and ileum (*p* < 0.001) compared to CONTR animals. Treatment with QCT (Group D) led to a significant twofold increase in cell proliferation in jejunum (*p* < 0.001) and ileum (*p* = 0.002) compared to MTX-nontreated animals (Group C). The proliferative zone in MTX rats moved progressively upward in the crypts toward the crypt-villus junction. At the same time, the proliferative zone of MTX-QCT rats was only mildly affected, showing a slight shift upward within the crypts.

### Enterocyte apoptosis

CONTR-QCT (Group B) demonstrated a significant increase in the rate of cell apoptosis compared to CONTR experimental group (Group A) (Fig. 3). MTX rats (Group C) demonstrated a strong increase in cell apoptosis in jejunum (*p* = 0.001) and ileum (*p* < 0.001) compared to CONTR animals. The AI decreased following QCT administration (Group D) in jejunum (*p* < 0.05) and ileum (*p* = 0.02) compared to MTX-untreated animals (Group C).

### Expression of proliferation and apoptosis-related proteins

**Fig. 4:**
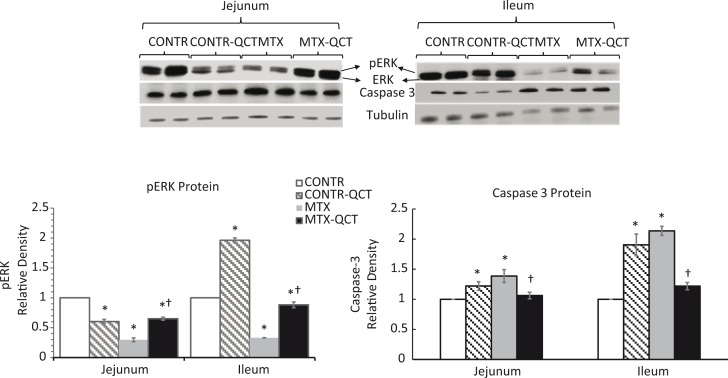
Effects of MTX and oral QCT on proliferation (p-ERK) and apoptosis (caspase-3)-related proteins (Western blot). Results are calculated as the ratio to tubulin and are expressed as a percentage of control animals. CONTR, control; MTX, methotrexate; QCT, quercetin. *p < 0.05 MTX and MTX-QCT versus CONTR rats; ^†^*p* < 0.05 MTX-QCT versus MTX.

A significant inhibition of cell proliferation in MTX rats (Group C) was accompanied by a significant decrease in p-ERK protein levels in jejunum (*p* < 0.05) and ileum (*p* < 0.05) compared to CONTR animals (Group A) (Fig. 4). MTX-QCT (Group D) rats demonstrated a significant increase in p-ERK protein expression in jejunum (*p* < 0.05) and ileum (*p* < 0.05) compared to MTX animals (Group C) that were correlated with a significant increase in cell proliferation rates in this group.

An increased cell apoptosis in MTX-treated rats (Group C) was associated with a significant elevation of caspase-3 protein levels in jejunum (*p* < 0.05) and ileum (*p* < 0.05) compared to CONTR animals (Group A) (Fig. 4). Treatment with QCT resulted in a significant decrease in caspase-3 protein levels in jejunum (*p* < 0.05) and ileum (*p* < 0.05) compared to MTX animals (Group A) that correlated with decreased cell apoptosis in this group.

## Discussion

Quercetin (QCT) is categorized as a flavonol, one of the five subclasses of flavonoid compounds that was found in leafy vegetables, many fruits, bulbs and tubers, herbs, spices, tea, and also wine (16, 17). These compounds are characterized by the presence of one or more phenol rings and two or more hydroxyl groups linked directly to the aromatic rings (18) and have been associated with antioxidant, anti-microbial, anti-proliferative, and anti-inflammatory properties (19–24). Polyphenols can mediate NF-kBp65 subunit translocation into the nucleus (25, 26) significantly modulating inflammatory cytokine secretion and we have recently reported that the inhibition of I kappa B kinase (IKK) phosphorylation is a crucial step in the cascade of events that starts with polyphenols exposure (27).

Several organs contribute to QCT metabolism, including the small intestine, the kidneys, the large intestine, and the liver, giving rise to glucuronidated, methylated, and sulfated forms of QCT; moreover, free QCT is also found in plasma. Unique biological elements of QCT contain potential mental and physical health benefits that include disease resistance; enhanced mental and physical performance; ability to inhibit lipid peroxidation; stimulation of mitochondrial biogenesis; as well as other anti-inflammatory, antiviral, and antioxidant properties (28, 29). Although QCT supplements have been promoted for the treatment of a wide spectrum of medical problems (30), the European Food Safety Authority found no cause-and-effect relationship for any physiological effect of QCT in human health or diseases (31). Recent experimental and clinical studies suggest a possible role for QRC in normal intestinal physiology and in several gastrointestinal disorders including NSAID-induced enteropathic damage and trinitrobenzene sulfonic acid (TNBS)–induced intestinal damage (10, 13, 32). QCT attenuates the clinical, morphological, and biochemical alterations induced by NSAID or TNBS possibly via its antioxidant mechanism.

In a recent randomized placebo-controlled double blind clinical trial, Kooshyar et al. have demonstrated that administration of 250mg QCT capsules twice daily for 4 weeks in 20 patients who underwent high-dose chemotherapy for blood malignancies resulted in a significant decrease in incidence of oral mucositis (33). The mechanisms of this positive effect were not investigated in this study.

In the current experiment, we investigated the effects of QCT on enterocyte turnover following MTX administration in *in vivo* rat model. Consistent with our previous experiments, treatment with MTX in the present study resulted in apparent intestinal damage. This conclusion is supported by the observed increase in injury score of MTX-treated rats compared to CONTR animals. MTX-treated rats presented with severe villous atrophy, epithelial flattening, and extensive crypt loss. In addition, treatment with MTX resulted in a significant mucosal hypoplasia. A decrease in bowel and mucosal weight, mucosal DNA and protein, and decrease in villus height support this conclusion. Parallel decreases in mucosal DNA and protein indicate that the smaller mucosal mass of MTX-treated animals can be attributed to cellular hypoplasia. MTX and its active metabolites block tetrahydrofolate synthesis by binding to the folic acid site on the enzyme dihydrofolate reductase. This action results in depletion of nucleotide precursors, and inhibition of DNA, RNA, protein synthesis, and cellular proliferation. Histologically, villus height decreased in response to MTX administration, suggesting decreased absorptive surface area; however, crypt depth decreased nonsignificantly. Analysis of epithelial proliferation 3 days after MTX administration, using BrdU incorporation as a marker, demonstrated an inhibition of DNA synthesis in the epithelium of the entire small intestine and concomitant decrease in the rates of cell proliferation. In addition, the proliferative zone in the crypts moved progressively upward toward the crypt-villus junction in MTX-treated rats. The mechanism responsible for this effect is poorly understood. Verburg and coworkers have shown in MTX-treated rats that BrdU-positive cells are not restricted to the crypts but are also found in up to one-third of the length of the villi due to migration of the cells (34).

We demonstrate that the observed decrease in enterocyte proliferation rate in MTX-treated animals was associated with decreased levels of p-ERK protein. The transmission of extracellular proliferation and differentiation signals into their intracellular targets is mediated by a signaling cascade culminating in mitogen-activated protein kinase (MAPK). MAPKs are serine-/threonine-specific protein kinases that regulate various cellular activities, such as gene expression, mitosis, cell proliferation, differentiation, and apoptosis. One of the MAPK signaling pathways triggered by cytokines or growth factors is the extracellular signal-related kinase (ERK) pathway.

Cell loss in the small intestine during MTX-induced mucositis is mainly regulated by programmed cell death. Apoptosis or programmed cell death is an active, genetically controlled process of cell suicide. It is a physiologic process whereby the body disposes of unwanted cells by self-destruction and is our utmost defense against damaged cells. Several studies on apoptosis focused on the role of the executioners, Cysteinyl-asparate-acid-proteinases, termed ‘caspases’ that are triggered in response to pro-apoptotic signals. Caspases cleave numerous substrates at the carboxyl side of an aspirate residue upon induction of apoptosis. A key caspase involved in the apoptotic pathway is caspase-3 (also known as Yama, CPP32, and Apopain). Inhibition of caspase-3 has been linked to prevention of apoptotic death *in vitro* (35), although certain stimuli can induce apoptosis by a caspase-3-independent pathway. In our study, elevated rates of cell apoptosis in MTX-treated rats was associated with a significant upregulation of caspase-3 levels compared to CONTR animals.

The present data clearly indicate that QCT has a strong stimulating effect on intestinal recovery from intestinal mucosal injury caused by MTX. Enteral administration of QCT in MTX-treated rats decreased the mucosal inflammation and reversed intestinal mucosal damage. This is evident from the decreased intestinal injury score. Although the mucosa was still severely damaged, MTX-QCT rats showed the presence of newly formed crypts and regeneration, less significant villous epithelial atrophy, and less significant polymorphonuclear leukocyte infiltration in the lamina propria. The mechanisms of this effect are unknown. The pathogenesis of chemotherapy-induced gastrointestinal mucositis has been described by Sonis et al. (36) and includes five phases: initiation by chemotherapy, upregulation and generation of messenger signals, signaling by pro-inflammatory cytokines and amplification of mucosal injury, ulceration of the mucosa, and finally healing. Several experiments evaluated the relationship between intestinal permeability, bacterial translocation, intestinal histology, and apoptosis during intestinal mucositis (37). Further experiments are required to determine whether positive effect of QCT in preventing intestinal damage is related to decreasing intestinal permeability as well as to decreased generation of pro-inflammatory cytokines (TNF-α, IL-1β, IL-6, IL-8), decreased activation of NFκB signaling, decreased leukocyte chemotaxis, and decreased T-cell reactivity, which were described in an animal model of inflammatory bowel disease.

In addition, reduced inflammation was coupled with enhanced intestinal repair. Increased bowel and mucosal weight as well as mucosal DNA and protein support this conclusion. Increased villus height may be the result of increased proliferation and accelerated migration along the villus and is a marker for the increased absorptive surface area and increased numbers of epithelial cells.

A significant stimulation of cell proliferation in both jejunum and ileum was demonstrated in MTX-QCT rats compared to MTX-nontreated animals, which was associated with elevated p-ERK protein levels that suggests activated stem cell activity and a stimulated MAPK signaling pathway. Li et al. have recently demonstrated that QCT promotes osteogenic proliferation and differentiation of mesenchymal stem cells through activation of the ERK1/2 and JNK signaling pathways (38). Lee and coworkers have shown that QCT enhances the proliferation of activated CD8(+) T cells by activating the TCF1/β-catenin axis via the PI3K/AKT/ERK pathway (39).

Increased mucosal proliferation in functioning intestine of MTX rats following QCT administration was accompanied by a decreased cell death via apoptosis that suggests an activated enterocyte turnover and may be considered as a major mechanism of mucosal hyperplasia in recovering bowel. Caspase-3 protein levels were downregulated in QCT-treated rats compared to MTX-non-treated animals that correlated with decreased rates of apoptosis. Although QCT has been shown to induce autophagy and apoptosis in various cancer cell types (9), several experiments have demonstrated anti-apoptotic effects of QCT in intestinal cells. Ben Salem et al. have shown that QCT treatment protect HCT116 and HEK293 cells from Zearalenone-induced apoptosis by reducing endoplasmic reticulum stress (40). Marchionatti and associates have demonstrated in 4-week-old chicks that QCT inhibits cell apoptosis by blocking the oxidative stress and by preventing FasL/Fas/caspase-3 pathway activation (41). Both elevated cell proliferation and decreased cell apoptosis in the current study may be considered as putative mechanisms responsible for compensatory hyperplasia and, thus, responsible for increased intestinal cell mass.

The mechanisms of the positive effects of QCT on intestinal mucosal homeostasis are not fully understood. Several experiments have demonstrated that exposure of animals to QCT prevents oxidative injury and cell death by various mechanisms including oxygen radical scavenging activity, inhibiting xanthene oxidase, lipid peroxidation, and chelating metal ions in experimental inflammatory bowel disease. Further experiments are required to investigate Herb–Drug interaction between QCT and MTX. Life-threatening interaction between MTX and the root extract of Pueraria lobate (42) and Rhubarb (the rhizome of Rheum palmatum L) (43) has been described recently in rats and could be a major hurdle in the success of a treatment in humans. This coadministration of herb and MTX may significantly change the elimination and result in markedly increased exposure to MTX in patients.

In conclusion, the use of QCT in rats given MTX prevents intestinal damage and enhances intestinal recovery. This appeared to occur via the induction of compensatory crypt cell proliferation and by inhibiting apoptosis. Although animal models do not always accurately replicate what happens in humans in a true clinical situation, this experiment provides important information about the possible beneficial role of QCT in improving treatment outcomes and quality of life for patients undergoing cancer treatment.
